# Associations between single-nucleotide polymorphisms of the interleukin-18 gene and breast cancer in Iraqi women

**DOI:** 10.5808/gi.22026

**Published:** 2022-06-30

**Authors:** Bilal Fadıl Zakariya, Asmaa M. Salih Almohaidi, Seçil Akilli Şimşek, Areege Mustafa Kamal, Wijdan H. Al-Dabbagh, Safaa A. Al-Waysi

**Affiliations:** 1 Department of Biology, Institute of Sciences, Çankiri Karatekin University, Çankırı Merkez 18100, Turkey; 2 Department of Biology, College of Science for Women, University of Baghdad, Baghdad 10022, Iraq; 3 Department of Biology, Institute of Sciences, Çankiri Karatekin University, Çankırı Merkez 18100, Turkey; 4 Department of Pathology/Oncology, Medical City Teaching Hospital, Baghdad 10011, Iraq; 5 Department of Oncology, Medical City Teaching Hospital, Baghdad 10011, Iraq; 6 Department of Microbiology, Medical City Teaching Hospital, Baghdad 10011, Iraq

**Keywords:** breast neoplasms, interleukin-18, polymorphisms, rs187238, rs1946518

## Abstract

According to long-term projections, by 2030, the world’s population is predicted to reach 7.5 billion individuals, and there will be roughly 27 million new cancer cases diagnosed. The global burden of breast cancer (BC) is expected to rise. According to the Ministry of Health-Iraqi Cancer Registry, cancer is the second largest cause of death after cardiovascular disease. This study investigated the interleukin-18 (*IL18*) single-nucleotide polymorphisms (SNPs) –607C/A rs1946518 and –137G/C rs187238 using the sequence-specific amplification-polymerase chain reaction approach. Regarding the position –607C/A, there was a highly significant difference between the observed and expected frequencies in patients and controls (*χ*^2^ = 3.16 and *χ*^2^ = 16.5), respectively. The AA and CA genotypes were associated with significantly increased BC risk (odds ratio [OR], 3.68; p = 0.004 and OR, 2.83; p = 0.04, respectively). Women with the A allele had a 5.03-fold increased susceptibility to BC. The C allele may be a protective allele against BC (OR, 0.19). Although position –137G/C showed no significant differences in the CC genotype distribution (p = 0.18), the frequency of the CC genotype was significantly higher in patients than in controls. In contrast, patients had a significantly higher frequency of GC genotypes than controls (p = 0.04), which was associated with an increased risk of developing BC (OR, 2.63). The G allele frequency was significantly lower in patients than in controls (55.0% vs. 76.2%, respectively). This SNP may be considered a common genotype in the Iraqi population, with the wild-type G allele having a protective function (OR, 0.19) and the mutant C allele having an environmental effect (OR, 2.63).

## Introduction

Breast cancer (BC) is the most common cancer in females worldwide, with nearly 2.3 million new cases diagnosed in 2020. It accounts for approximately 11.7% of all cancer cases and 24.5% of all cancer cases in females [1]. Since 2008, the global incidence of BC has increased by more than 20%, and the death rate has risen by 14% [2]. As a result, research on relevant tumor indicators for early diagnosis and monitoring is required, and recent studies have concentrated on the role of the immune system in cancer progression [3].

BC is a multi-step process that involves numerous genetic changes, such as oncogene activation and cancer suppressor gene inactivation [4]. The gene encoding interleukin (IL)-18 is found on chromosome 11 at positions 11q22.2–q22.3 and has six exons in humans. IL-18 is a multidirectional cytokine that regulates the immune response in various ways; in 1989, IL-18 was first described as an “interferon-inducing factor.” IL-18 plays an essential role in stimulating natural killer cells, and cellular anticancer activities also enhance the expansion of Th1 and cell activation. Furthermore, IL-18 increases the expression of adhesion-related molecules, nitric oxide synthase enzyme synthesis, and chemokine production [5]. IL-18, in combination with IL-2, causes a Th2 cell response and the production of IL-4 and IL-13. Simultaneously, IL-18 can reduce antitumor immunity in a programmed-death-1 (PD-1) dependent manner. PD-1 is a co-inhibitory receptor that constitutes one of the top checkpoints. Many polymorphisms in the *IL-18* promoter region affect transcript factor binding locations [6], which could be *IL-18* expression quantitative trait loci. Several studies have found that cytokine gene polymorphisms impact cytokine production, which may be linked to disease [7].

Single-nucleotide polymorphisms (SNPs) are found in the *IL18* gene, particularly in the promoter region bound to –607G/T (rs1946518) and –137G/C (rs187238). These SNPs (–137G/C and –607G/T) in the *IL18* gene promoter region are expected to affect IL-18 expression and activity [8]. Arimitsu et al. [9] found that monocytes in individuals with the 137G/G genotype produced considerably more *IL-18* than those in individuals with the 137G/C genotype. Furthermore, a link between these two SNPs and cancer susceptibility has been established [10]. Research on the relevance of *IL18* polymorphisms in BC risk remains contentious. To our knowledge, only three studies have examined the influence of *IL18* genetic variations (–137G/C and –607A/C) on BC susceptibility; Khalili-Azad et al. [11] studied the effect of *IL18* polymorphisms (–607A/C and –137G/C) on BC risk in 200 patients and 206 healthy controls. They discovered that CC homozygosity for the –137G/C polymorphism lowered the risk of BC [11]. Other studies found that the *IL18* –607A/C polymorphism contributed to an increased risk of BC [12,13]. In light of this information, it is necessary to investigate *IL18* SNPs in Iraqi women with BC.

## Methods

After the ethical committee of the Medical City directorate approved this study, 100 women who visited the oncology teaching hospital's breast clinic were recruited from January 28, 2020 to August 11, 2022. Group 1 included 60 women who had recently been histopathologically diagnosed with BC and provided written informed consent for participation in the study. Group 2 included 40 healthy women who served as controls. Patients with other systemic diseases and those taking any hormone-modifying drug were excluded from the study.

### DNA extraction and genotyping

Each patient and control had 8-10 mL of blood taken. Blood samples were taken from the cubital vein and placed directly into an anticoagulant tube containing EDTA. Genomic DNA extraction was performed using a Norgen Biotek kit (Thorold, ON, Canada). The optical density ratio at 260/280 nm was used to assess the quantity and quality of isolated DNA Using a Nano-Drop device (Qubit 4, Invitrogen, Waltham, MA, USA). It was preferable to have a balance of 1.7–1.9. Electrophoresis on a 1% agarose gel was performed to corroborate the findings. Until the genotyping analysis, the DNA samples were stored at –20°C.

### Polymerase chain reaction

Polymorphisms were investigated by utilizing sequence-specific amplification-polymerase chain reaction (SSP-PCR) at locations –607C/A rs1946518 and –137G/C rs187238 within the promoter region of *IL18*. For the targeted location –607C/A, a common reverse primer and two specific forward primers were utilized, with an amplified product size of 196 bp, and a forward control primer was used to amplify a 301-bp fragment covering the polymorphism region as an internal control (Table 1).

In the location –137G/C, a common reverse primer and two specific forward primers were also utilized, with an amplified product size of 261 bp, and a forward control primer was used to amplify a 446-bp fragment covering the polymorphism region as an internal control (Table 2). The polymerase chain reaction (PCR) reactions were carried out in a volume of 25 μL, including 12.5 μL of 2× Go-Taq Green Master mix (Promega, Madison, WI, USA), 3 μL of genomic DNA, and 6.5 μL of nuclease-free water. All reaction mixtures contained one sequence-specific primer, one common reverse primer, and one internal position control primer, each at a concentration of 1 μL. As a result, each piece of DNA was subjected to two PCR tests: one for the F1 wild-type allele and one for the F2 mutant allele [8]. The assays were carried out in a thermocycler (Applied Biosystems, Waltham, MA, USA). Denaturation was carried out at 95°C for 5 min, followed by 30 cycles at 94°C for 30 s, 57°C for 30 s, 72°C for 40 s, and 72°C for 10 min. Then, the PCR results were visualized using electrophoresis on 2% agarose gel, and the molecular weight was calculated using a 100-bp DNA ladder.

### Statistical analysis

The genotype of cytokines was statistically analyzed using the SPSS version 25 (IBM Corp., Armonk, NY, USA). The allele frequencies of the cytokine genes were calculated by the direct gene counting method. A freely available online calculator was used to calculate whether there was a significant departure from Hardy-Weinberg (H-W) equilibrium for two alleles (http://www.had2know.com/academics/Hardy-Weinberg equilibrium-calculator-3-alleles.html). H-W equilibrium is the expected frequency of genotypes if mating is non-assortative and there are no mutations from one allele to another. When there are two alleles for a particular gene, A and B, and their respective population frequencies are p and q, the expected frequencies of the genotypes AA, AB, and BB are p^2^, 2pq, and q^2^, respectively. The Pearson chi-square test was used to assess whether there were significant differences between the observed and expected frequencies. The alleles and genotypes of cytokines were presented as percentages and frequencies, and the two-tailed Fisher exact test was calculated to assess the significance of differences between their distributions in BC patients and controls. Odds ratios (ORs) were also estimated to define the association between cytokine alleles and genotypes with BC. OR values can range from between 0 and 1 (for a negative association) to more than 1 (for a positive association).

## Results

### *IL-18* –607C/A (rs1946518) SSP-PCR

**Fig. 1.**
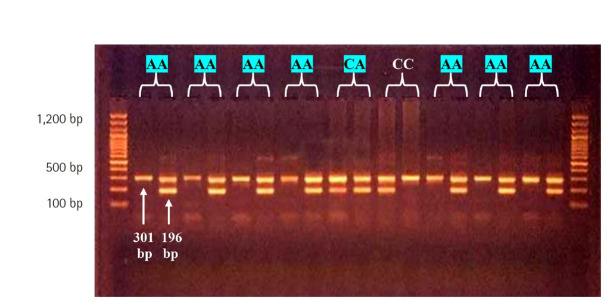
Agarose gel electrophoresis image that shows the sequence-specific amplification-polymerase chain reaction product analysis of interleukin-18 (*IL18*) –607 (rs1946518) (C/A) gene polymorphism. Where M, marker (100–1,200 bp), the presence of C or A allele were observed at 196-bp product size. The (CC) wild type homozygote were showed in C allele only, the (AA) mutant type homozygote were showed in A allele only, whereas the (C/A) heterozygote were showed in both C and A allele, internal control at 301-bp product size.

This study analyzed the distribution of the genotype and allele frequencies of the rs1946518 polymorphism (at the –607C/A position) in patients and controls (Fig. 1). This polymorphism presented three genotypes (CC, CA, and AA) that corresponded to two alleles (T and A) in BC patients and controls. The genotype frequencies in both groups were not in agreement with H-W equilibrium, and there were highly significant differences between the observed and expected frequencies in both patients and controls (*χ*^2^ = 3.16 and *χ*^2^ = 16.5), respectively (Table 3).

The CC genotype was significantly less common in patients than in controls (15.0% vs. 65.5%, p < 0.001), and the magnitude of this negative association was 0.11. However, the AA genotype frequency was higher in patients (51.7%) than in controls (22.5%); this difference was significant (OR, 2.83; p = 0.004). The CA genotype frequency was also significantly higher in patients than in controls (33.3% vs. 15.0%; OR, 3.68; p = 0.04). The C allele frequency was lower in patients than in controls (25.0% vs. 38.7%), while the A allele frequency was higher (75.0% vs. 61.3%, respectively). A negative association was found for the C allele (OR, 0.19) and a positive association for the A allele (OR, 5.03) (p < 0.001 for both) (Table 4).

### *IL-18*–137G/C (rs187238) SSP-PCR

**Fig. 2.**
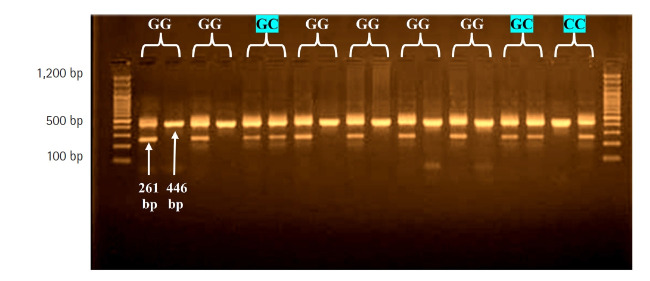
Agarose gel electrophoresis image that shows the sequence-specific amplification-polymerase chain reaction product analysis of interleukin-18 (*IL18*) –137 (rs187238) (G/C) gene polymorphism. Where M, marker (100–1,200 bp), The presence of G or C allele were observed at 261-bp product size. the (GG) wild type homozygote were showed in G allele only, the (CC) mutant type homozygote were showed in C allele only, whereas the (G/C) heterozygote were showed in both G and C allele. internal control at 446-bp product size.

The rs187238 genetic polymorphism of the *IL18* gene was investigated at position –137G/C (Fig. 2), and presented with three genotypes (GG, GC, and CC) that corresponded to two alleles (G and C). The results of patients were in agreement with the expected H-W equilibrium. However, in the control group, the results were not in agreement with the expected (H-W) equilibrium, and there was a highly significant difference between the observed and expected frequencies (*χ*^2^ = 5.74) (Table 5).

At the position –137G/C in *IL18*, patients had a significantly higher frequency of the GC genotype than controls (43.3% vs. 22.5%; OR, 2.63; p = 0.04). In contrast, the frequency of the CC genotype was significantly higher in patients than in controls (23.3% and 12.5%; OR, 2.13). Therefore, the G allele frequency was significantly lower in patients than in controls (55.0% vs. 76.2%, respectively), with a highly significant difference (p = 0.002). A negative association was found for the G allele (OR, 0.38) and a positive association for the C allele (OR, 2.36) (Table 6).

## Discussion

The present study investigated the two most commonly studied SNPs of the *IL18* gene (–607C/A and –137G/C). The patients and controls both showed deviation from H-W equilibrium for the -607C/A genotype, which may have been related to BC or intermarriage in Arab Iraqi society between relatives. This result aligns with that of a previous study on BC [14].

Although the distribution of the –137G/C genotype in the patient group was consistent with H-W equilibrium, this was not the case for the control group, which showed a highly significant difference between the observed and expected frequencies (*χ*^2^ = 5.74). This SNP may be considered a common genotype in the Iraqi population, with the wild-type G allele having a protective function, reducing susceptibility, and the mutant C allele having an environmental effect.

These SNPs' genotypes and alleles showed significant differences between BC patients and controls. The present study observed that the *IL18* –607 CA and AA genotypes were present in about 85% of BC cases. The wild-type CC genotype had a low frequency (about 15%) in BC patients, which may highlight the role of the *IL18* –607 polymorphism in the pathogenesis of disease. The A allele was an environmental effect allele, while the C allele had a preventive fraction because the CC genotype showed the highest frequency in the control group (70%). These highly significant findings for the AA and CA genotypes at position *IL18* –607 suggest that this polymorphism may play a role in cancer progression.

Several studies on *IL18* polymorphisms have been conducted in various populations in multiple countries; one of them has found a link between *IL18* polymorphisms and the risk of BC [15]. The *IL18* –607C/A polymorphism may be linked to an increased risk of BC in Asian and mixed populations [16]. Furthermore, the present results showed that the *IL18* rs1946518 SNP might play a role in BC because the wild-type allele C of the –607 SNP had a protective effect against BC (OR, 0.19). In contrast, the mutant allele A had a positive association (OR, 5.03) suggesting an etiological impact; therefore, women who carry allele A of –607 may be more susceptible to BC than women who have allele C. However, the results of some studies were mixed; in a study involving 72 BC patients and 93 control women, Fathi Maroufi et al. [17] discovered that the *IL18* -607A/C polymorphism was not linked to BC in an Iranian population sample. The *IL18* rs187238 polymorphism results showed no significant differences according to the CC genotype frequencies between BC patients and controls. Nonetheless, it was interesting to note in the present study that the heterozygous GC genotype had an OR of 2.63 for the patient group, implying that the mutant allele C may have had an environmental effect on the Iraqi population, conferring susceptibility to BC, while the wild-type allele G had a preventive effect against BC (OR, 0.38). Our findings show that the *IL18* -137G/C polymorphism is associated with the development of BC. The conversion of G (guanine) to C (cytosine) at position -137G/C of the *IL18* gene removes a nuclear factor binding site for histone-4 transcriptional factor-1 [18]. Genetic variants have been considered the most critical cancer risk factors. Although high-penetrant capability genes (e.g., *BRCA1* and *BRCA2*) have strong links to BC, low-penetrant susceptibility genes that predispose individuals to the disease have yet to be identified; nonetheless, immune responses and surveillance may be affected by genetic variability in a sequence of immune regulatory genes [19]. The *IL18* promoter polymorphism –137G/C has previously been linked to various cancers in different populations, including esophageal squamous cell malignant tumors, prostate cancer in the Chinese population [20], colorectal cancer in Greek people [21], and ovarian cancer in native Hawaiians [22]. Additional case-control studies on BC and gastric cancer progression have been published [23,24]. However, there is no link between type 2 diabetes mellitus development and the *IL18* –137G/C gene polymorphism [25].

## Figures and Tables

**Table 1.** Primers for the *IL18* ‒607C/A rs1946518 polymorphism

	Primer sequence
Forward primer 1	5′-GTTGCAGAAAGTGTAAAAATTATTA**C**-3′
Forward primer 2	5′-GTTGCAGAAAGTGTAAAAATTATTA**A**-3′
Reverse primer	5′-TAACCTCATTCAGGACTTCC-3′
Internal positive control	5′-CTTTGC TATCATTCCAGGAA-3′

*IL18*, interleukin-18.

**Table 2.** Primers for the *IL18* ‒137G/C, rs187238 polymorphism

	Primer sequence
Forward primer 1	5′-CCCCAACTTTTACGGAAGAAAA**G**-3′
Forward primer 2	5′-CCCCAACTTTTACGGAAGAAAA**C**-3′
Reverse primer	5′-AGGAGGGCAAAATGCACTGG-3′
Internal positive control	5′-CCAATAGGACTGATTATTCCGCA-3′
	

*IL18*, interleukin-18.

**Table 3.** Hardy-Weinberg equilibrium–expected genotype frequencies in *IL18* rs1946518

Groups	CC	CA	AA	*C*	*A*	*χ* ^2^
Patient						
genotypes						
Observed	9	20	31	0.68	0.32	3.16^a^
Expected	6.0	26.0	28.0	Not detected		-
Control						
genotypes						
Observed	25	6	9	0.30	0.70	16.5^a^
Expected	19.6	16.8	3.6	Not detected		-

*IL18*, interleukin-18.

a *χ*^2^ > 3.84, significant.

**Table 4.** Genotype distribution and allele frequency of *IL18* rs1946518 (‒607C/A) in breast cancer patients and controls with risk estimation

	Study group		OR (95% CI)	Fisher’s exact probability^*^	p-value
	Patient (n = 60)	Control (n = 40)			
Genotype groups					
C/C	9 (15.0)	25 (62.5)	0.11 (0.04–0.28)	0.000	0.0001^***^
C/A	20 (33.3)	6 (15.0)	2.83 (1.02–7.86)	0.061	0.040^*^
A/A	31 (51.7)	9 (22.5)	3.68 (1.49–9.04)	0.003	0.004^**^
Allele distribution					
*C*	38 (31.7)	56 (70.0)	0.19 (0.11–0.36)	0.000	0.0001^***^
*A *	82 (68.3)	24 (30.0)	5.03 (2.72–9.30)	0.000	

*IL18*, interleukin-18; OR, odds ratio; CI, confidence interval. Significant,

* p < 0.05,

** p < 0.01,

*** p < 0.001.

**Table 5.** Hardy-Weinberg equilibrium–expected genotype frequencies in *IL18* rs187238

Group	GG	GC	CC	*G*	*C*	χ^2^
Patients’						
genotypes						
Observed	20	26	14	0.45	0.55	0.93 NS
Expected	18.2	29.7	12.2	Not detected		-
Control						
genotypes						
Observed	26	9	5	0.24	0.76	5.74^a^
Expected	23.3	14.5	2.30	Not detected		-

*IL18*, interleukin-18; NS, not significant.

a *χ*^2^ > 3.84, significant.

**Table 6.** Genotype distribution and allele frequency of *IL18* rs187238 (‒137G/C) in breast cancer patients and controls with risk estimation

	Study group		OR (95% CI)	Fisher’s exact probability^*^	p-value
	Patient (n = 60)	Control (n = 40)			
Genotype groups					
G/G	20 (33.3)	26 (65.0)	0.27 (0.12–0.62)	0.002	0.002^**^
G/C	26 (43.3)	9 (22.5)	2.63 (1.07–6.48)	0.035	0.04^*^
C/C	14 (23.3)	5 (12.5)	2.13 (0.70–6.47)	0.203	0.180 ^NS^
Alleles distribution					
*G*	66 (55.0)	61 (76.2)	0.38 (0.20–0.71)	0.002	0.002^**^
*C*	54 (45.0)	19 (23.8)	2.63 (1.40–4.92)	0.03	

*IL18*, interleukin-18; OR, odds ratio; NS, non-significant. Significant,

* p < 0.05,

** p < 0.01.
